# Which Is More Valuable in the Diagnosis of Pulmonary Thromboembolism? The Wells Score, the Revised Geneva Score, or the Padua Score?

**DOI:** 10.3390/life15071115

**Published:** 2025-07-16

**Authors:** Hasan Veysel Keskin, Neslihan Ozcelik, Elvan Senturk Topaloglu, Songul Ozyurt, Aziz Gumus, Unal Sahin

**Affiliations:** Department of Pulmonology, Faculty of Medicine, Recep Tayyip Erdogan University, 53100 Rize, Türkiye; keskinh.veysel@gmail.com (H.V.K.); neslihan.ozcelik@erdogan.edu.tr (N.O.); songul.ozyurt@erdogan.edu.tr (S.O.); aziz.gumus@erdogan.edu.tr (A.G.); unal.sahin@erdogan.edu.tr (U.S.)

**Keywords:** pulmonary embolism, clinical probability scoring, Wells, Geneva, Padua

## Abstract

Background: Pulmonary thromboembolism (PTE) is a preventable yet potentially fatal condition with significant morbidity and mortality. Several clinical scoring systems, including the Wells and modified Geneva scores, have been developed to assess the likelihood of PTE and guide further diagnostic evaluation. The Padua prediction score, primarily used to assess venous thromboembolism (VTE) risk in hospitalized patients, has also been considered for its potential utility in suspected PTE cases. Methods: This retrospective study included 257 patients with suspected acute PTE. Diagnosis was confirmed by computed tomography pulmonary angiography (CTPA) in 140 patients (patient group), while 117 patients without radiologic evidence of PTE served as controls. All participants were evaluated using Wells, modified Geneva, and Padua scores. Sensitivity, specificity, predictive values, and the effect of combining scores with age-adjusted D-dimer levels were analyzed. Results: The Wells score demonstrated a sensitivity of 60% and specificity of 91%, with a positive predictive value of 88%. Modified Geneva and Padua scores showed lower diagnostic accuracy. Negative predictive values increased significantly when combined with age adjusted D-dimer levels. Conclusions: The Wells score was the most reliable tool among the three for predicting PTE. Combining clinical scoring with D-dimer testing enhances diagnostic accuracy and may reduce unnecessary imaging in patients with low to moderate risk.

## 1. Introduction

The mean annual incidence of venous thromboembolism (VTE) ranges from 75–269/100,000, whereas pulmonary thromboembolism (PTE), representing a major clinical presentation of VTE, occurs at a rate of 39–115/100,000 annually [1,2,3]. The incidence of VTE is gradually increasing as a result of the increase in the number of people at risk in aging societies with prolonged life expectancy and the higher sensitivity of diagnostic imaging methods [4,5]. The 30 day mortality and 7 day mortality due to PTE were found to be 1.8% and 1.1%, respectively [6]. In patients with PTE, the mortality risk is particularly high in those who do not receive adequate treatment and increases up to 53% in patients who have never received anticoagulant therapy for various reasons. Mortality is 3.3% in patients who have started treatment but did not complete 90 days of treatment [7]. Based on these data, the most important factor to prevent mortality in PTE is early initiation and receiving adequate treatment.

The first step in the diagnosis of pulmonary thromboembolism is suspicion. In patients with risk factors such as immobilization, history of cancer, trauma and major surgery, PTE should be suspected in the presence of complaints such as sudden shortness of breath, chest pain, hemoptysis, syncope or presyncope. PTE should be suspected especially in patients with dyspnea and tachycardia, with a normal chest X-ray and no other disease that could explain the current symptoms. Clinical symptoms and signs can vary from asymptomatic to sudden shock and death, depending on the size and localization of the embolus, whether an infarction develops, and whether the embolus is recurrent or not. This variability depends on the age of the patient, the reserve of cardiopulmonary functions, the localization of the embolism, the development of the infarction and the rate of resolution [8]. The absence of specific symptoms makes diagnosis difficult. Chest X-ray, electrocardiography (ECG), complete blood count, biochemical tests and arterial blood gas (ABG) are not sufficient to confirm the diagnosis but are useful for clinical evaluation and to rule out other causes. Pulmonary angiography, once considered the gold standard for diagnosing PTE, has been replaced by computed tomography pulmonary angiography (CTPA), which has been shown to have similar diagnostic accuracy.

Predicting the risk of PTE by looking at risk factors in addition to the patient’s symptoms and signs allows for both a further differentiation of patients for further investigation and an initiation of treatment without wasting time. For this reason, clinical probability scores have been established to assist and guide clinicians. The most widely known of these and the most commonly used are the Wells (Canada) scoring and the modified Geneva scoring systems [9,10].

Another scoring system, the Padua Score, is used to decide whether a hospitalized patient should receive prophylaxis for VTE. It was developed as a result of a prospective observational study conducted with 1180 hospitalized patients and consists of 11 risk factors [11].

Patients with a total score of four and above are considered high risk. Primary thromboprophylaxis is recommended in high-risk patients. In this study, 711 of 1180 patients were found to be low risk (<4 points) and only two low-risk patients developed VTE during follow-up when primary thromboprophylaxis was not administered [11]. In a study conducted on 1067 patients to evaluate the value of the Padua score in predicting the diagnosis of PTE, it was observed that high Padua score values were associated with positive CTPA. In addition, in this study, it was found that four predictive values could discriminate between positive and negative CTPA [12].

To our knowledge, no prior study has comprehensively compared the diagnostic performance of the Padua score with both the Wells and modified Geneva scores in patients undergoing diagnostic evaluation for acute PTE.

The aim of our study was to evaluate the superiority and drawbacks of the Padua, Wells and modified Geneva clinical scores in predicting PTE in patients undergoing further investigation for suspected PTE and to compare their effectiveness.

## 2. Patients and Methods

### 2.1. Patient Groups and Collection of Data

Patients aged 18 years and older who were evaluated for suspected acute pulmonary thromboembolism (PTE) between June 2019 and November 2021 were retrospectively included in this study. The clinical and laboratory variables required to calculate the Wells, modified Geneva, and Padua scores were extracted from electronic medical records recorded at the time of initial patient evaluation.

Patients were assessed using two distinct classification frameworks. First, they were categorized into the following two diagnostic groups based on computed tomography pulmonary angiography (CTPA) findings: those with confirmed PTE and those without. A positive diagnosis was defined as the presence of a filling defect in segmental or more proximal pulmonary arteries on CTPA. Subsegmental emboli were not considered diagnostic and were therefore excluded.

Second, patients were stratified according to their pre-test clinical probability using the Wells, modified Geneva, and Padua scores. Based on the established thresholds of each scoring system, patients were grouped as either “likely PTE” or “unlikely PTE.” This dual classification approach enabled evaluation of the diagnostic performance of clinical scores and comparison with actual imaging-confirmed outcomes.

Patients who were unable to undergo CTPA due to contraindications such as contrast allergy or renal failure, those with missing laboratory data at admission, and those under 18 years of age were excluded from the study.

For all included patients, demographic characteristics, presenting symptoms, clinical risk factors, comorbidities, initial vital signs, and laboratory results were recorded. In addition, available findings from compression Doppler ultrasonography (CDU) and transthoracic echocardiography (ECHO) were documented. The Wells, modified Geneva, and Padua scores were calculated for each patient accordingly.

### 2.2. Clinical Scoring Systems

In this study, the following three validated clinical probability scoring systems were used to assess the likelihood of pulmonary thromboembolism: the Wells score, the revised Geneva score, and the Padua prediction score. The Wells score incorporates both objective findings and a subjective clinical judgment regarding the likelihood of alternative diagnoses, and it categorizes patients into low, moderate, or high probability groups. The revised Geneva score consists exclusively of objective criteria and stratifies patients similarly. The Padua score, originally designed for assessing the risk of venous thromboembolism in hospitalized medical patients, includes 11 clinical variables and is primarily used to guide decisions on thromboprophylaxis. All three scoring systems were calculated for each patient using the clinical and demographic data recorded at admission. The thresholds for categorization were applied as defined in the original scoring models (Table 1).

### 2.3. Statistical Analysis

Statistical evaluations were performed using the IBM-SPSS program (SPSS version 21; SPSS Inc., Chicago, IL, USA). The conformity of continuous variables to normal distribution was analyzed by Kolmogorov–Smirnov test. Continuous variables were expressed as mean ± standard deviation and minimum–maximum, and categorical variables were expressed as %. In the comparison of two groups, Student’s *t*-test was used for variables showing normal distribution and Mann–Whitney U test was used for variables not showing normal distribution. A chi-square test was used in the comparison of categorical variables. A value of *p* < 0.05 was considered statistically significant. ROC (receiver operating characteristic) analysis was performed to determine the effect of clinical probability scores in differentiating pulmonary embolism from normal patients. In addition, specificity, sensitivity, positive predictive value and negative predictive values of clinical probability scores were calculated.

## 3. Results

The study included 257 patients with suspected acute PTE. A total of 140 patients whose diagnosis was confirmed by CTPA were classified as the “patient group” and 117 patients without PTE diagnosis were classified as the “control group”.

The median age of the patients in the patient group was 74 (19–99). The median age of the control group patients was 71 (19–97) and there was no significant difference between the groups (*p* = 0.269). The admission symptoms, risk factors and comorbidities of the patients are shown in Table 2.

The most common admission symptoms in the patient group were dyspnea in 114 (81.4%) patients, chest pain in 41 (29.2%) patients and syncope in 29 (20.7%) patients. In the control group, the most common admission symptoms were dyspnea in 92 (78.6%) patients, bloody sputum in 18 (15.3%) patients and chest pain in 15 (12.8%) patients (Table 2).

In the PTE group, the most prevalent risk factors were immobility (n = 85, 60.7%), recent hospitalization within the past three months (n = 46, 32.8%), a history of deep vein thrombosis (DVT) or pulmonary thromboembolism (PTE) (n = 32, 22.8%), and active malignancy (n = 29, 20.7%). Similarly, in the control group, the most common risk factors included immobility (n = 45, 38.4%), hospitalization in the past three months (n = 41, 35%), and malignancy (n = 37, 31.6%) (Table 2).

Hypertension and malignancy were the most frequently reported comorbidities in both groups, with comparable distributions between the PTE group (hypertension: n = 69, 49.2%; malignancy: n = 29, 20.7%) and the control group (hypertension: n = 61, 43.5%; malignancy: n = 37, 26.4%) (Table 2).

Table 3 shows the patients’ physical examination findings and laboratory parameters at admission. The median D-dimer value was calculated to be 4300 µg/mL (250–10,000) in the patient group and 1235 µg/mL (200–8590) in the control group and was found to be statistically significant (*p* < 0.001).

According to the clinical scoring methods, 88% of the patients classified as “likely PTE” were diagnosed with PTE by Wells method, 61% by modified Geneva method and 61% by Padua method. In patients classified as “unlikely PTE”, PTE was diagnosed by Wells, modified Geneva and Padua methods as 34%, 43% and 41%, respectively.

It was observed that D-dimer values were significantly higher in the patient group classified as “likely PTE” according to clinical scoring methods than in the patient group classified as “unlikely PTE” (Table 4).

Among the patients’ group, the scoring that classified most patients as “likely PTE” was Padua scoring with a rate of 76%, while this rate was calculated as 60% and 69% in Wells and modified Geneva scoring, respectively. In the control group patients, the scoring that classified most patients as “unlikely PTE” was Wells scoring with a rate of 91%, while this rate was calculated as 48% and 41% in Padua and modified Geneva scoring, respectively (Table 5).

**Figure 1:**
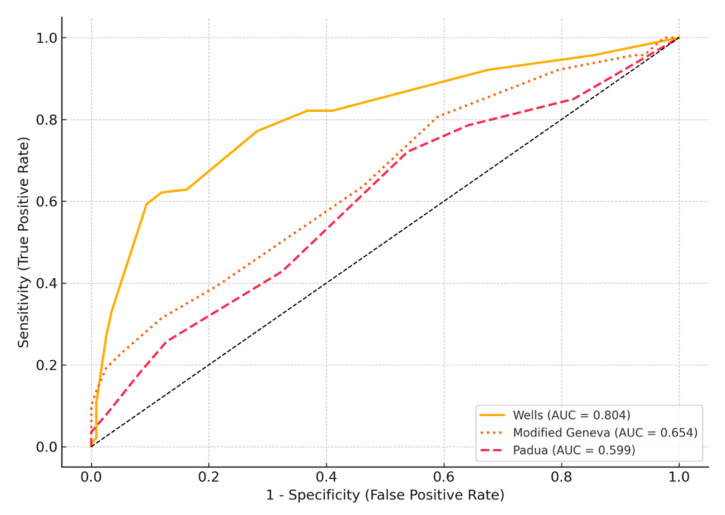
ROC curves of clinical probability scoring (Statistical comparison of AUCs using DeLong’s method yielded *p*-values as follows: Wells vs. Geneva, *p* < 0.001; Wells vs. Padua, *p* < 0.001; Geneva vs. Padua, *p* = 0.047).

ROC curves were constructed and the areas under the curve were evaluated to determine the differential (threshold) values of Wells, modified Geneva and Padua scoring upon reaching the diagnosis and to compare them with each other (Figure 1). It was determined that the area under the curve was the highest in Wells scoring, followed by modified Geneva, and therefore these two methods were more valuable than Padua method in reaching the diagnosis (AUC for Wells: 0.811, AUC for modified Geneva: 0.659, AUC for Padua: 0.606). Statistical comparison of AUCs using DeLong’s method yielded *p*-values as follows: Wells vs. Geneva, *p* < 0.001; Wells vs. Padua, *p* < 0.001; Geneva vs. Padua, *p* = 0.047.

The sensitivities, specificities, positive predictive values and negative predictive values of Wells, modified Geneva and Padua scoring obtained in our study are shown in Table 6.

**Figure 2:**
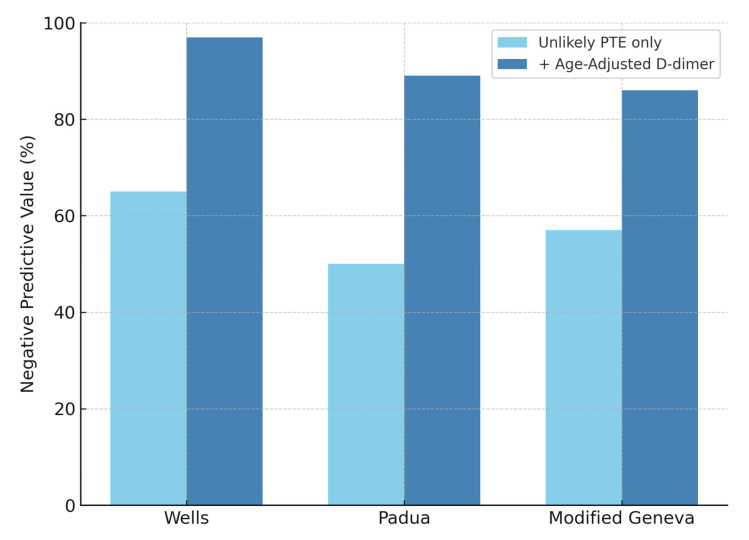
Negative predictive value (NPV) improvements after adding age-adjusted negative D-dimer to “unlikely PTE” classification based on Wells, Padua, and Modified Geneva scores.

In our study, when the cases classified as “unlikely PTE” by clinical probability scoring were combined with “age-adjusted negative D-dimer”, the negative predictive values increased from 65% to 97% for Wells and from 50% to 89% for Padua and from 57% to 86% for modified Geneva (Figure 2).

## 4. Discussion

In this study, we compared the diagnostic accuracy of the Padua, Wells, and modified Geneva clinical probability scores for acute pulmonary embolism (PTE). Among the three, the Wells score exhibited superior discriminative ability, demonstrated by the highest area under the ROC curve (AUC). Moreover, when complemented with an age-adjusted negative D-dimer in patients classified as “unlikely PTE” the Wells score showed a marked enhancement in negative predictive performance. This supports the hypothesis that integrating a clinical likelihood assessment with laboratory data may improve diagnostic efficiency in suspected PTE.

The Wells score outperformed both the Padua and modified Geneva scores in our cohort in terms of AUC, specificity, and positive predictive value (PPV). This indicates its superior capacity to correctly identify true PTE cases while minimizing false positives. One possible explanation for this advantage is the inclusion of subjective clinical judgment in the Wells criteria—most notably, the item “alternative diagnosis less likely than PTE.” When administered by experienced clinicians, this element appears to enhance diagnostic accuracy. In contrast, the modified Geneva score, though fully objective and reproducible, may lack sensitivity to clinical subtleties encountered in real-world settings. Additionally, the Padua score was originally designed to estimate venous thromboembolism risk in hospitalized patients rather than to diagnose symptomatic PTE in emergency scenarios. These factors help explain the consistently stronger diagnostic performance of the Wells score in our study and align with previous literature emphasizing its clinical utility.

When patients classified as having an “unlikely probability” of pulmonary thromboembolism (PTE) based on clinical scoring systems were re-evaluated in conjunction with age-adjusted negative D-dimer results, the negative predictive values (NPVs) of all three models improved, with the Wells score achieving an NPV of 97%. These results suggest that incorporating D-dimer testing into clinical assessment strategies may enhance the ability to safely exclude PTE, particularly in low-risk populations.

Several studies in the literature have compared the diagnostic accuracy of the Wells and Geneva scores. In a meta-analysis by van Es et al., including 7268 patients with suspected PTE, the sensitivity and specificity of the Wells score were reported as 53% and 79%, respectively, with an AUC of 0.73 (95% CI: 0.72–0.75) [13]. Another study prospectively comparing the Wells and Simplified Modified Geneva scores found a sensitivity of 70% and specificity of 78% for the Wells score, while the Geneva score demonstrated 66% sensitivity and 72% specificity. The AUCs were 0.85 (95% CI: 0.81–0.89) and 0.76 (95% CI: 0.71–0.80), respectively (*p* = 0.005) [14]. Similarly, in a study by Wong et al., the specificity of the Wells score (67.5%, 95% CI: 57.4–77.6) was significantly higher than that of the modified Geneva score (47.0%, 95% CI: 36.3–57.7; *p* = 0.002) [15]. A separate meta-analysis evaluating both scores reported ranges of 63–79% for sensitivity and 48–90% for specificity of the Wells score, with an AUC of 0.778 (95% CI: 0.740–0.818). The modified Geneva score showed a sensitivity of 55–73%, specificity of 51–89%, and an AUC of 0.693 (95% CI: 0.653–0.736) [16].

In our study, the sensitivity and specificity of the Wells score were calculated as 60% and 91%, respectively, with an AUC of 0.793. For the modified Geneva score, sensitivity was 69%, specificity was 48%, and the AUC was 0.697. These findings align with previous studies, which further supports the higher diagnostic specificity and overall performance of the Wells score in identifying PTE compared to the modified Geneva score.

In a study by Di Marca et al., which evaluated the applicability of the Wells and modified Geneva scores in hospitalized older adults with multiple comorbidities, the AUC values were reported as 0.79 (95% CI: 0.67–0.91) for the Wells score and 0.71 (95% CI: 0.58–0.84) for the modified Geneva score, with a statistically significant difference (*p* < 0.001) [17]. Similarly, Guo et al. reported positive predictive values (PPV) of 83.8% and 61.3% for the Wells and modified Geneva scores, respectively, and negative predictive values (NPV) of 85% and 80%. The AUC was 0.823 (95% CI: 0.710–0.976) for the Wells score and 0.661 (95% CI: 0.631–0.983) for the modified Geneva score [18].

In our study, the AUC values observed for the Wells and modified Geneva scores were consistent with those reported in the aforementioned studies. Specifically, the AUC was 0.811 (95% CI: 0.758–0.864) for the Wells score and 0.659 (95% CI: 0.593–0.725) for the modified Geneva score, both of which were statistically significant (*p* < 0.001 for both). Similarly, the PPV for the Wells and modified Geneva scores in our cohort was 88% and 61%, respectively, closely aligning with previously published data. However, the observed NPVs of 65% and 57% were lower than those reported in prior studies. It has been widely documented that the combination of a negative D-dimer result with a low clinical probability score provides a safe strategy to rule out PTE [19,20,21,22]. For instance, Wells et al. demonstrated that among 437 patients with a low clinical probability and a negative D-dimer result, only one case of PTE was detected, corresponding to an NPV of 99.5% (95% CI: 99.1–100%) [9].

In our cohort, when clinical scores classified as “unlikely PTE” were combined with age-adjusted negative D-dimer results, the NPVs improved considerably: 97% for the Wells score, 89% for the Padua score, and 86% for the modified Geneva score. These findings indicate that the integration of clinical probability assessment with age-adjusted D-dimer may enhance the safety of excluding PTE in low-risk populations.

The Padua score was originally developed by Barbar et al. as a risk assessment model for venous thromboembolism (VTE) in hospitalized medical patients [11]. Kandagatla et al. later evaluated the predictive value of the Padua score specifically for PTE [12]. In their study involving 1067 patients, the AUC was reported as 0.64 (95% CI: 0.60–0.69, *p* = 0.046), with sensitivity and specificity values of 57.3% and 66.8%, respectively. The PPV and NPV were 22.6% and 88.2% [12]. In line with these findings, the Padua score in our study showed a sensitivity of 47% and specificity of 63%, with an AUC of 0.606 (95% CI: 0.537–0.674; *p* = 0.004).

A key distinguishing feature of the Wells score compared to the modified Geneva and Padua scores is the inclusion of the item “an alternative diagnosis is less likely than PTE”, which introduces a subjective clinical judgment component. While both the modified Geneva and Padua scores consist solely of objective criteria, the Wells score has been subject to debate due to this subjective element, which may lead to inter-clinician variability [12,15,23]. Notably, previous literature has shown that this subjective criterion contributes significantly to the total Wells score and may substantially influence its diagnostic performance [24]. Despite this variability, our findings and those in the literature consistently demonstrate that the Wells score yields higher sensitivity and specificity compared to more objective scoring systems. This suggests that clinician judgment—when appropriately applied—remains a critical factor in the diagnostic evaluation of suspected PTE.

Although numerous studies have compared the diagnostic performance of the Wells and modified Geneva scores, our study provides a preliminary evaluation that also includes the Padua score within the same analytical framework. Consistent with existing literature, the Wells score demonstrated the highest diagnostic performance, particularly in terms of specificity and positive predictive value. While the Padua score reached statistical significance in predicting PTE, its lower sensitivity, specificity, and AUC limited its clinical utility in this setting.

### Limitations

This study has several limitations. First, it was conducted at a single center with a relatively small sample size, which may limit the generalizability of the findings. Additionally, clinical scores were calculated retrospectively from medical records, which may have introduced documentation-related bias. Future prospective, multicenter studies with larger cohorts are warranted to validate and extend these results.

## 5. Conclusions

In conclusion, our findings support the Wells score as the most effective clinical tool for diagnosing pulmonary thromboembolism (PTE) among the scoring systems evaluated. Its diagnostic performance was further enhanced when combined with an age-adjusted negative D-dimer result, significantly improving its negative predictive value. These results highlight the clinical value of integrating structured clinical assessment with laboratory data in the diagnostic work up of suspected PTE.

## Figures and Tables

**Table 1:** Clinical decision rules of the Wells, modified Geneva and Padua scores.

Score Points	
Wells Score *	
Symptoms and signs of DVT	3
Alternative diagnosis less likely than PTE	3
Tachycardia (>100 beats/min)	1.5
Immobilization or surgery in the last 4 weeks	1.5
History of DVT or pulmonary embolism	1.5
Hemoptysis	1
Cancer Presence	1
* Greater than 2: low clinical probability; 2 to 6: moderate clinical probability; greater than 6: high clinical probability.	
Modified Geneva Score **	
Previous history of DVT or PTE	3
Age > 65 years	1
Surgery or extremity fracture within 4 weeks	2
Hemoptysis	2
Unilateral lower limb pain	3
Presence of active cancer	2
Heart rate 75–94 beats/min	3
Heart rate > 95 beats/min	5
Pain on lower limb deep vein palpation and unilateral edema	4
** Zero to 3: low clinical probability; 4 to 10: moderate clinical probability; greater than or equal to 11: high clinical probability	
Padua Score ***	
Active cancer ^a^	3
History of venous thromboembolism	3
Immobilization ^b^	3
Known thrombophilic disease ^c^	3
Recent (within the last 1 month) trauma and/or operation	2
Advanced age (>70 years)	1
Heart and/or respiratory failure	1
Acute myocardial infarction/ischemic stroke	1
Acute infection and/or rheumatic disease	1
Obesity (body mass index > 30 kg/m^2^)	1
Hormone replacement therapy	1
*** Low risk of VTE < 4, High risk of VTE ≥ 4	

^a^ Patient with local or distant metastasis and/or receiving chemo/radiotherapy in the last 6 months. ^b^ Patient who will or is expected to fulfill all his/her needs in bed for at least 3 days. ^c^ Antithrombin, protein C or S deficiency, factor V Leiden, G20210A prothrombin mutation, antiphospholipid syndrome.

**Table 2:** Clinical and demographical data of the patients.

Variable	PTE Group (*n* = 140)	Control Group (*n* = 117)	*p*-Value
**Age, median (min–max)**	74 (19–99)	71 (19–97)	0.269
**Gender (Male/Female)**	90/50	54/63	0.004
**Symptoms, *n* (%)**			
Shortness of breath	114 (81.4%)	92 (78.6%)	0.576
Chest pain	41 (29.2%)	15 (12.8%)	0.001
Syncope	29 (20.7%)	5 (4.3%)	<0.001
Change in consciousness	25 (13.0%)	13 (11.1%)	0.129
Leg swelling	21 (15.0%)	8 (6.8%)	0.547
Hemoptysis	11 (7.8%)	18 (15.3%)	0.058
**Risk Factors, *n* (%)**			
Immobility	85 (60.7%)	45 (38.4%)	<0.001
Hospitalization (last 3 months)	46 (32.8%)	41 (35.0%)	0.712
History of DVT/PTE	32 (22.8%)	2 (1.7%)	<0.001
Active malignancy	29 (20.7%)	37 (31.6%)	0.046
Recent surgery (last 3 months)	12 (8.5%)	7 (5.9%)	0.430
Recent trauma (last 1 month)	10 (7.1%)	1 (0.85%)	0.013
Long-term travel	6 (4.2%)	2 (1.7%)	0.236
Pregnancy	2 (1.4%)	0 (0%)	0.194
**Comorbidities, *n* (%)**			
Hypertension	69 (49.2%)	61 (43.5%)	0.649
Malignancy	29 (20.7%)	37 (26.4%)	0.046
Cerebrovascular disease	23 (16.4%)	10 (7.1%)	0.060
Coronary artery disease (CAD)	20 (14.2%)	13 (9.2%)	0.449
Alzheimer’s disease	23 (16.4%)	10 (7.1%)	0.056
Diabetes mellitus (DM)	19 (13.5%)	21 (15.0%)	0.335
Congestive heart failure	13 (9.2%)	12 (8.5%)	0.794
Atrial fibrillation (AF)	13 (9.2%)	20 (14.2%)	0.062
Asthma	13 (9.2%)	3 (2.1%)	0.026
Chronic obstructive pulmonary disease (COPD)	11 (7.8%)	16 (11.4%)	0.130

Note: Malignancy is listed under both “Risk Factors” and “Comorbidities” due to differing definitions; under Risk Factors, it refers to active cancer at admission; under Comorbidities, it includes both active and historical malignancy.

**Table 3:** Physical examination and laboratory data of the patients.

	PTE Group	Control Group	
	n = 140 Median (Min–Max)	n = 117 Median (Min–Max)	*p* Value
Pulse	92 (50–160)	89 (60–150)	0.391
Systolic Blood Pressure (SBP)	120 (70–190)	120 (70–200)	0.398
Diastolic Blood Pressure (DBP)	70 (37–120)	80 (50–100)	0.162
Fever	36.6 (36.1–38.6)	36.6 (36.2–38.4)	0.876
Respiratory Rate (RR)	20 (16–40)	18 (16–36)	0.009
Arterial blood gas pH	7.43 (7.13–7.60)	7.41 (7.20–7.54)	0.007
PCO_2_ (mm/Hg)	32 (21–63)	43 (19–91)	<0.001
PO_2_ (mm/Hg)	59 (44–108)	59 (45–119)	0.706
SO_2_	90 (72–99)	90 (75–99)	0.363
Lactate (mmol/L)	1.6 (0.5–6.9)	1.2 (0.4–6.5)	0.018
Haemogram	9 (2.8–28)	9.5 (2.9–27.8)	0.327
WBC (10^3^/uL)	12 (7–16.7)	12.7 (7.2–16.9)	0.552
HGB (g/dL) PLT (10^3^/uL)	208 (50–548)	239 (45–666)	0.002
Other	0.82 (0.3–7.1)	0.88 (0.35–8)	0.233
Creatine (mg/dL)	18.1 (0–348)	64 (0.2–400)	<0.001
CRP (mg/L)	4300 (250–10,000)	1235 (200–8590)	<0.001
D-dimer(µg/mL) Troponin (µg/L)	0.04 (0.003–1810)	0.03 (0.003–1513)	0.012

Pulse: beats per minute (bpm), SBP and DBP: mmHg, Respiratory rate (RR): breaths per minute, SaO_2_: percentage (%), WBC: White Blood Cell, HGB: Hemoglobin concentration, PLT (10^3^/µL): Platelet count, CRP: C-reactive protein.

**Table 4:** Comparison of D-dimer values in “unlikely PTE” and “likely PTE” patients according to Wells, modified Geneva and Padua scores.

	“PTE Unlikely” D-Dimer in Patients Median (Min–Max)	“PTE Likely” D-Dimer in Patients Median (Min–Max)	*p* Value
Wells	1690 (200–10,000)	3760 (250–10,000)	<0.001
Modified Geneva	1855 (200–10,000)	2870 (200–10,000)	0.028
Padua	1770 (200–10,000)	2880 (250–10,000)	0.023

**Table 5:** Distribution of “likely PTE” and “unlikely PTE” patients according to Wells, modified Geneva and Padua scores in PTE group and control group.

Scoring System	PTE Group n = 140	Control Group n = 117	*p* Value
Wells Scoring	Likely (>4): 84 (60%) Unlikely (≤4): 56 (40%)	Likely (>4): 11 (9%) Unlikely (≤4): 106 (91%)	0.001
Modified Geneva Scoring	Likely (≥6): 97 (69%) Unlikely (<5): 43 (31%)	Likely (≥6): 61 (52%) Unlikely (<5): 56 (48%)	0.005
Padua Scoring	Likely (>4): 107 (76%) Unlikely (≤4): 33 (24%)	Likely (>4): 69 (59%) Unlikely (≤4): 48 (41%)	0.003

**Table 6:** Sensitivity, specificity, PPV and NPV rates of clinical probability scoring.

Test	Sensitivity % (95% CI)	Specificity % (95% CI)	PPV % (95% CI)	NPV % (95% CI)
Wells	60 (51.7–67.7)	91 (83.9–94.7)	88 (80.4–93.4)	65 (57.8–72.3)
Padua	47 (39.1–55.4)	63 (54.2–71.4)	61 (51.2–69.2)	50 (42.0–58.0)
Modified Geneva	69 (61.2–76.3)	48 (39.0–56.8)	61 (53.6–68.6)	57 (46.7–65.9)
D-Dimer	97 (92.9–98.9)	34 (26.2–43.2)	56 (57.2–70.0)	92 (78.8–96.4)

Sensitivity, specificity, PPV and NPV are shown with their 95% confidence intervals (CI), PPV: Positive predictive value, NPV: Negative predictive value.

## Data Availability

All data generated or analyzed during this study are included in this article. The data will be available upon reasonable request (contact person: elvan.senturktopaloglu@erdogan.edu.tr).
